# The complete mitochondrial genome of Blue-winged Minla (*Minla cyanouroptera*) and its phylogenetic analysis

**DOI:** 10.1080/23802359.2019.1659109

**Published:** 2019-08-29

**Authors:** Weiqiang He, Huailiang Xu, Diyan Li, Meng Xie, Mingwang Zhang, Qingyong Ni, Yongfang Yao

**Affiliations:** a College of Life Science, Sichuan Agricultural University, Ya’an, China;; b College of Animal Science and Technology, Sichuan Agricultural University, Chengdu, China

**Keywords:** Mitochondrial genome, Blue-winged Minla, phylogenetic analysis

## Abstract

Blue-winged Minla is a small-sized bird, belonging to the Family Leiothrichidae of the Passeriformes. The population is suspected to be in decline owing to ongoing habitat destruction and fragmentation. Therefore, we sequenced and analyzed the Blue-winged Minla mitochondrial genome to provide a theoretical basis for protecting the brids. The mitochondrial genome of Blue-winged Minla was 17862 bp, it contains 13 protein-coding genes, 22 transfer RNA genes, 2 ribosomal RNA genes, and 2 control regions. The overall base composition of the mitochondrial was 30.88% A, 13.82% G, 23.86% T, 31.44% C.

Blue-winged Minla (*Minla cyanouroptera*) is a small-sized bird, belonging to the Family Leiothrichidae of the Passeriformes. The length of body is 13–16 cm, similar in gender, grey-brown overhead with light blue and black stripes. Blue-winged Minla natural habitat is subtropical or tropical moist montane forests and it is distributed in Bhutan, Cambodia, China, India, Lao People’s Democratic Republic, Nepal, Thailand, Viet Nam. Despite the fact that the population trend appears to be decreasing, the decline is not believed to be sufficient rapid to approach the thresholds for Vulnerable under the population trend criterion. Therefore, the species is evaluated as Least Concern (BirdLife International 2016). The population is suspected to be in decline owing to ongoing habitat destruction and fragmentation. Therefore, we sequenced and analyzed the Blue-winged Minla mitochondrial genome to provide a theoretical basis for protecting the brids.

The sample was collected from Ya’an (30°0′47.20″N, 103°02′25.51″E) Sichuan province of China and Stored at the Sichuan Agricultural University Museum, China and sample ID was 9092.xc Total DNA of Blue-winged Minla was isolated from muscle tissue by the traditional phenol-chloroform method (Sambrook 2001). Fifteen pairs of primers were used to amplify the overlapping segment of its mitochondrial genomic by the Polymerase Chain Reaction (PCR). Then splicing sequences and analyzed complete mitochondrial genome by software DNAstar (Burland 2000). We have submitted the complete mitochondrial sequences of Blue-winged Minla to GeneBank and got the accession number: MK779708.

The complete mitochondrial genome of Blue-winged Minla published in this paper, and mitochondrial DNA is 17862 base pairs in length, contained 13 protein-coding genes, 2 ribosomal RNA genes, 22 transfer RNA genes, and 2 control regions (Dloop1, Dloop2) which was similar to other mitochondrial genome of Passeriformes (Xue et al. 2016; Liu et al. 2018). The overall base composition of the genome was 30.88% A, 13.82% G, 23.86% T, 31.44% C. All tRNA genes possess the typical clover leaf secondary structure except for tRNA^ser^ which lack arm of dihydrouracil (DHU). *NADH6* and eight tRNA genes were transcribed from the L-strand, the rest genes of Blue-winged Minla were encoded on the H-strand. All protein-coding genes used ATG as start coding, except for *NADH6* which began with CTA, there are two types stop codon which complete or incomplete, 6 PCGs (*NADH1*, COXII, *ATPase8*, *ATPase6*, *NADH4L*, *CYTB*) shared stop codon TAA, 2 PCGs (*NADH2*, *NADH6*) shared stop codon CAT, COXI and NADH5 stopped with AGG, AGA, 3 PCGs shared incomplete codon. Consistent with previous research results, two ribosomal RNA genes were located between the tRNA^phe^ and tRNA^Leu^ which separated by the tRNA^Val^ (Zhang 2015; Weiqiang He et al. 2018).

We downloaded eight whole mitochondrial sequence of Passeriformes from the NCBI, and *Melopsittacus undulates* was used as the outgroup (Figure 1). Using the MEGA10.0 for neighbor-joining (NJ) methods to construct a phylogenetic tree (Kumar et al. 2018). The phylogenetic tree suggested that nine species clustered into two clades, including Leiothrichidae and Sylviidae. Other than this, we found that Mina cyanouroptera and Mina ignotincta have close relationship.

**Figure 1.**
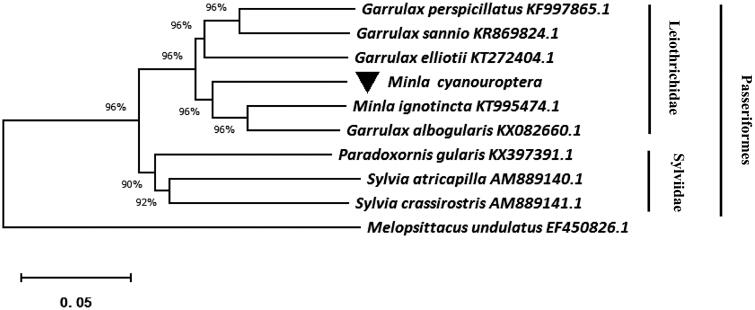
Neighbor-joining (NJ) tree based on nine species whole mitochondrial sequence, and *Melopsittacus undulates* was used as the outgroup. Black inverted triangle represented a sequence of this study.
